# Measuring intratumor heterogeneity by network entropy using RNA-seq data

**DOI:** 10.1038/srep37767

**Published:** 2016-11-24

**Authors:** Youngjune Park, Sangsoo Lim, Jin-Wu Nam, Sun Kim

**Affiliations:** 1 Interdisciplinary Program in Bioinformatics, Seoul National University, Seoul, 151-742, Korea; 2 Department of Life Science, College of Natural Sciences, Hanyang University, Seoul, 133-791, Korea; 3 Research Institute for Natural Sciences, Hanyang University, Seoul, 133-791, Korea; 4 Department of Computer Science and Engineering, Seoul National University, Seoul, 151-742, Korea; 5 Bioinformatics Institute, Seoul National University, Seoul, 151-742, Korea

## Abstract

Intratumor heterogeneity (ITH) is observed at different stages of tumor progression, metastasis and reouccurence, which can be important for clinical applications. We used RNA-sequencing data from tumor samples, and measured the level of ITH in terms of biological network states. To model complex relationships among genes, we used a protein interaction network to consider gene-gene dependency. ITH was measured by using an entropy-based distance metric between two networks, nJSD, with Jensen-Shannon Divergence (JSD). With nJSD, we defined transcriptome-based ITH (tITH). The effectiveness of tITH was extensively tested for the issues related with ITH using real biological data sets. Human cancer cell line data and single-cell sequencing data were investigated to verify our approach. Then, we analyzed TCGA pan-cancer 6,320 patients. Our result was in agreement with widely used genome-based ITH inference methods, while showed better performance at survival analysis. Analysis of mouse clonal evolution data further confirmed that our transcriptome-based ITH was consistent with genetic heterogeneity at different clonal evolution stages. Additionally, we found that cell cycle related pathways have significant contribution to increasing heterogeneity on the network during clonal evolution. We believe that the proposed transcriptome-based ITH is useful to characterize heterogeneity of a tumor sample at RNA level.

Cancer has a complex system consisting of different cancer clones that interact with each other and also with normal cells, known as intratumor heterogienty (ITH)^1^. The complexity from ITH is a major hurdle to understanding of the dynamics of cancer systems and also difficult to predict therapeutic outcomes^2^. Intratumor heterogeneity is the consequence of clonal evolution of a single tumor^3^. One of the main cause of this ITH is genomic instability of cancer cells^4^. High-throughput sequencing technology is widely used to measure ITH at molecular level. A recent study revealed that diverse clones with different genomic signatures co-exist in a single tumor^5^. Diversity of clones give evolutional advantage in metastasis^6^. Additionally, diverse subclones are known to be under high pressure of natural selection in therapeutic circumstance and even cause therapeutic resistance^7^,^8^. This clonal evolution during chemotherapy makes current target-drug therapy difficult^2^,^9^,^10^. However, there still remains an evolutionary issue about selective process during neoplasia, i.e., which daughter cells are selected and survive. To this issue, a colon cancer study suggested a big bang model without selective sweeps and a liver cancer study proposed non-darwinian evolution in tumor^11^,^12^. Whether or not selective force being present, overproduction of subclones highly-likely results in ITH.

Molecular level ITH has been identified with multiregional sequencing^11^,^13^. Although this multiregional sequencing is at the forefront of ITH studies, single-cell genomics has emerged as the most credible technology^14^. Single-cell sequencing has an advantage on direct sequencing of each clone^15^. However, experimental cost of single-cell sequencing is too high for clinical applications. Thus, researchers have developed computational methods to infer ITH with bulk-tumor sequencing data as an aggregated metadata of each clone’s genomic information. In general, daughter cells carry exactly the same parental genomic information. However, their DNA replication system malfunctions, often in cancer, and leaves *de novo* mutational signatures, furthermore copy number alterations (CNA) and loss of heterozygosity (LOH)^16^,^17^. Those genomic alterations remain from generation to generation, thus enabling the backtracing genomic signatures^13^,^18^. On the same principle, inferring subclones from the genomic landscape of bulk tumor sequencing is a widely used strategy^19^,^20^,^21^. Computational methods, such as PyClone and EXPANDS, are current state-of-the-art tools that use mutational information to infer subclonal populations^22^,^23^. Clinical relevance of inferred ITH was also highlighted in related to prognostic outcomes^24^,^25^.

Although the ITH inference based on genomic information were successful, there remain a few more issues that need further investigation. For example, a study reported that patients with a moderate number of subclones (3 or 4 clones) implicates a higher risk than more heterogeneous patients (above 4 clones)^24^. They discussed that there is a trade-off between the advantage of diversity and the cost of generating inviable daughter cells, however as mentioned earlier the selective sweep during cancer progression is still in questions. To understand better in tumor heterogeneity and clonal evolutionary process, we need to investigate three issues when genomic information is used for ITH prediction. First, it is a difficult to define whether a somatic mutation as either a driver or a passenger mutation in terms of cancer genome evolution^26^. The study about neutral evolution of tumor proposed that driver mutation can be altered differently in a certain context^27^. As a result, inference of ITH with driver gene mutations may not reflect true subclonal population. Second, the mutational information alone is insufficient to identify cellular activities of subclones in cancer. Furthermore, cell plasticity needs to be considered in ITH since phenotypes of cancer subclones can be altered without inheritable genomic variations^28^. A colon cancer study revealed that different phenotypes can exist with no differences in genotypes^29^. Lastly, cancer microenvironment is important in clonal evolution, tumor progression and metastasis^1^,^30^,^31^. According to current researches, different clonal activities and surrounding stromal and immune cells effects on cancer progression^32^,^33^. This finding was also confirmed in a single-cell sequencing study^34^. However, mutational lineage analysis could only detect heterogeneity of cancer clones, not other effects from microenvironmental factors. Therefore, we believe that, in addition to the current DNA-based ITH inference, measuring ITH at the RNA level can provide a new insight on ITH and its clinical applications.

To investigate the functional differences of heterogeneous clones, we developed a method for ITH inference using RNA-sequencing data. There are two major reasons why RNA-sequencing data was used. First, RNA- sequencing data is ubiquitous as much as genomic data. Second, like mutations, transcriptome is also used in evolutionary studies^35^,^36^. However, there is a challenge for analysis at the RNA level. Complex gene-gene dependency needs to be considered^37^. Thus, we used a biological network which is the most effective tool for modeling the complex gene-gene relationship - protein interaction network (PIN) and pathway information^38^,^39^,^40^.

Given a network, an effective metric is needed to quantify differences in the network perturbation to reflect both expression levels of individual genes and their relationships such as network topology and also co-expression of genes. We used an information theoretic approach to measure network state. This approach was successful in measuring network perturbations in terms of gene expressional changes^41^,^42^,^43^. This entropy measure was also effective for detecting network state transition from the normal state to the disease state^44^,^45^. A recent application of the network entropy successfully showed the difference between primary tumor and metastatic tumor^46^. Additionally, Signaling entropy studies by Teschendorff group identified relationship between network entropy and differentiation potential, additionally the prognostic importance^47^,^48^,^49^.

Our hypothesis is that a heterogeneous tumor will have more ambiguity in network than a homogeneous one (Fig. 1). Thus, we developed a novel measurement of ITH with transcriptome data using information theory, network-based Jensen-Shannon Divergence (nJSD)^50^. Our approach was extensively tested for issues related with ITH. For proof of concept, we used human cancer cell line data and single cell sequencing data. Then, the pan-cancer cohort data was analyzed. Our result was in agreement with widely used genome-based ITH inference methods. Additionally, our approach was also tested for immune cell infiltration. Finally, analysis of mouse clonal evolution showed that our network perturbation inference was consistent with ITH at different clonal evolution stages.

## Methods

### Calculation of nJSD

Jensen-Shannon Divergence is the measure similar to Kullback-Leibler divergence with some modifications to make JSD symmetric and bounded in a finite value^51^. nJSD is the sum of entropy values measured at each of the genes in a protein interaction network. To define entropy of each gene, it is necessary to define a probability distribution using gene expression values. We used log2-normalized gene expression values and assumed that the protein interactions were under the law of mass action.

Let *e*_*i*_ denotes the expression level of gene-*i* and a set of neighbor genes of gene-*i* is *J*_*i*_. Then, a probability of interaction between two genes is defined as


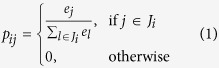


With *p*_*ij*_, we defined a probability distribution of gene-*i* which has 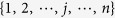 neighbors on PIN on a sample *X*.


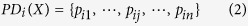


Let the *l*^*th*^-element in the probability distribution *PD*_*i*_(*X*) be *PD*_*i*_(*X*)_*l*_, then the Kullback-Liebler Divergence of gene-*i* between normal (*N*) and tumor (*T*) is defined^52^.


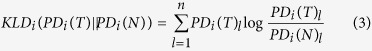


Then the JSD of gene-*i* between normal and tumor was defined as


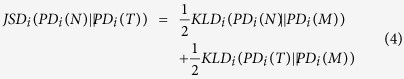


where 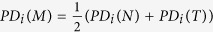.

Finally, nJSD was defined as an average JSD of all genes. Graphical example of calculation of nJSD was described in (Supplementary Fig. 1).

### Calculation of transcriptome-based ITH

**Figure 2:**
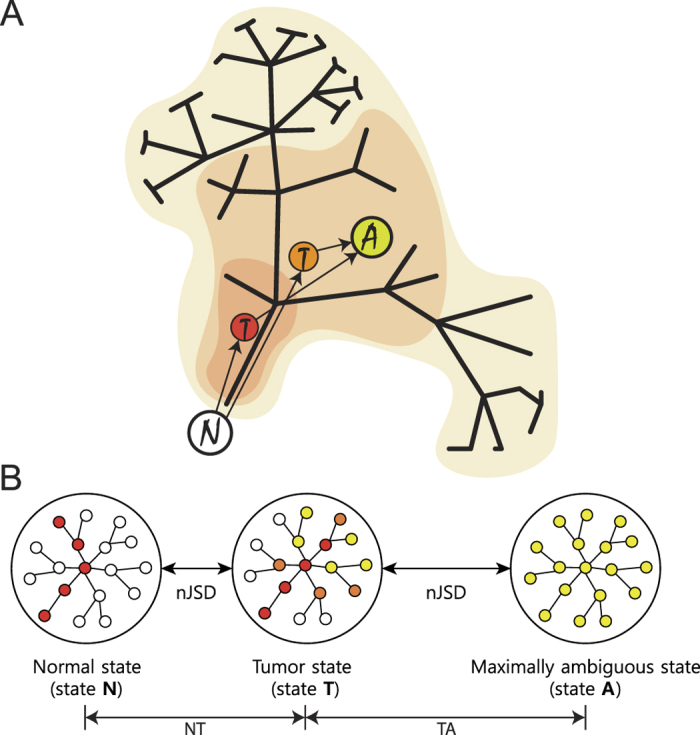
Overproduction during clonal evolution made ambiguous network status. (**A**) The consequence of clonal evolution is single tumor with heterogeneous population of cancer clones. The red shade which has smallest area on darwin’s tree would be early cancer and it’s sequencing result is represented as red circled ‘T’. Orange one has larger area than red one, of course, orange one has more diverse population. Lime one has the most diverse population. We set a maximal state of ambiguous like lime one, most diverse population of cancer clones, and measured tITH. (**B**) Network represents tumor with diver population. Distance between each state measured with nJSD, described in Method.

To define transcriptome-based ITH (**tITH**), we set a maximally ambiguous network where whole gene-expression values were equal. nJSD was applied as a distance measure between two network states. Here, we defined tITH with two distance values, distance from normal data to cancer data (*NT*) and distance from cancer data to maximally ambiguous network (*TA*) (Fig. 2). This distance based approach was inspired by recent study about cancer evolution that described embryonic stem cell as cancer evolutionary destination^53^. Combining *NT* and *TA* into a single metric, we defined the transcriptome-based ITH and we named it as tITH in comparison with genomic ITH (gITH).


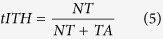


To investigate tITH at the pathway level, **pathway**-**tITH** was defined using only the gene set in a specific pathway. With this metric, we were able to precisely quantify pathway perturbation value ranging from 0 to 1.

## Results

### Proof of concept of state A with *in silico* simulated data and single-cell sequencing data

To calculate tITH, we assumed a maximally ambiguous state (state A). We made *in silico* data to investigate the relationship between sample heterogeneity and network ambiguity using single-cell sequencing data.

**Figure 3:**
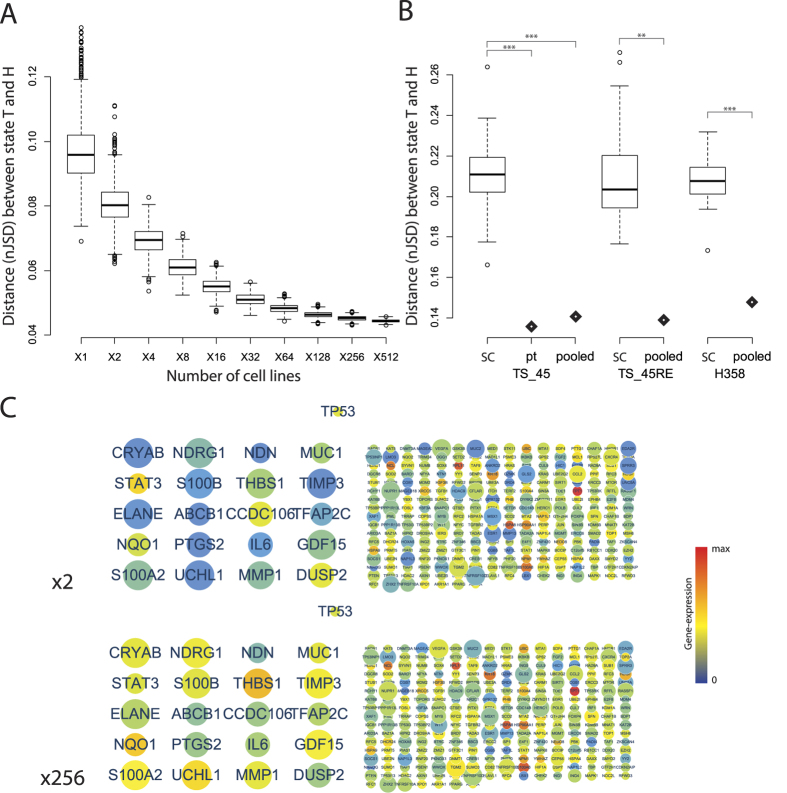
Heterogeneous sample show ambiguous network state like state A. (**A**) is result of *in silico* mixed data with 675 human cancer cell lines. (**B**) is result of real bio-data from single-cell sequencing study. The bulk tumor sequencing data is more closer to state A than each of single-cell data in three different LUAD data set. “SC” represents single-cell data, “pt” represents patients derived tumor data and “pooled” represents pooled tumor cell data. Z-score test was performed. (**C**) Protein-interaction network of *TP53* gene and its neighbors of *in silico* mixed data, X2 and X256. We highlighted top 20 genes in terms of difference between two conditions. Other gene names could be found in Supplementary Fig. 2.

Each of cancer cell lines of different molecular characteristics such as drug resistance was cultured in well-controlled environments, so as to consider each cell line as different clone in heterogeneous tumor. With 675 different human cancer cell lines, we created heterogeneous tumor gene-expression data and calculated distance to state A by nJSD. The distance to state A decreased as more cell lines were mixed (Fig. 3A). This indicates that data from bulk-cell sequencing of tumor with diverse clones will show ambiguous network state similar to the state A. The difference in network status can be easily identified by visualizing the networks with gene expression values (Fig. 3C). In a plot of the gene expression value vs. the number of genes at a certain expression level, shape of the line in the cell line mixed data shifting to the one in the state A from the skewed shape in individual cell lines (Supplementary Fig. 3).

The relationship between sample heterogeneity and network ambiguity was re-examined with single-cell sequencing data as a real biological data. Diverse mutational patterns among different clones could contribute to differentially activated paths in network across clones, thus we expect that the bulk-cell tumor data will show more ambiguous network status than each of single-cell data. The LUAD data set consists of 3 different experimental sets (H358, LCT-PT-45 and LCT-PT-45Re with 35, 44 and 50 single-cell sequencing, respectively and additional pooled cell sequencing data)^54^. We compared patient derived bulk tumor and pooled sample with its single-cell sequencing data in three different LUAD data set. nJSD of 10,051 genes were calculated and compared in terms of distance from state A. Patient derived bulk tumor, “pt”, was significantly closer to state A than single cells in TS_45 data (*Z*-score = −4.01, p-value = 0.00003) (Fig. 3B). Pooled sample also had lower nJSD to state A than single cells in H358, LCT_PT_45 and LCT_PT_45Re data (*Z*-score = −5.27 and p-value < 0.00001, *Z*-score = −3.75 and p-value = 0.000087 and *Z*-score = −3.08 and p-value = 0.001042).

With *in silico* simulated data and single-cell sequencing data, it was possible to identify that the heterogeneous sample had ambiguous network state than homogeneous one. Our tITH measurement would showed how their gene-expression profile changed from normal tissue to maximally ambiguous state using nJSD.

### tITH showed comparable result with gITH

This experiment is to show how well tITH agrees with gITH, genomic information based ITH, using the pan-cancer data set from TCGA. To compare tITH with gITH inferred clonal information was obtained from the study using PyClone and EXPAND^24^. Since different mutational pattern will influence network perturbations genetic heterogeneity ought to be associated with tITH^55^,^56^.

**Figure 4:**
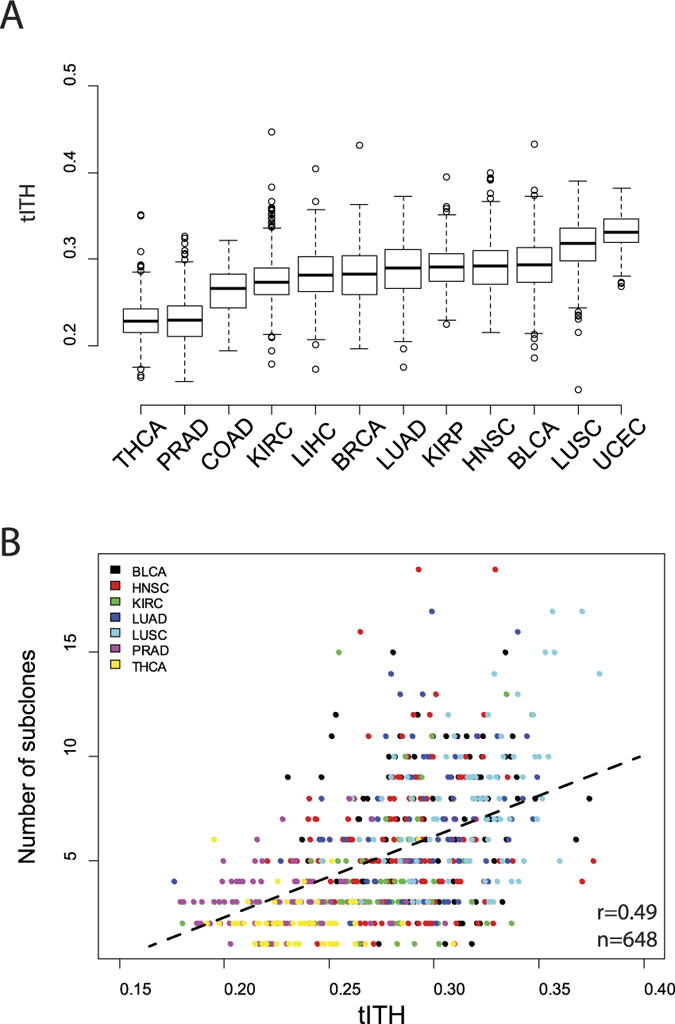
TCGA pan-cancer data and ITH. (**A**) Boxplot shows that the inter-tumor types differences of tITH distribution. (**B**) tITH and the number of subclones is positively correlated. 648 patients in 7 different cancer types are analyzed (*R*^2^ = 0.24, p-value < 2.2e-16).

tITH of pan-cancer cohort, 5,630 patients, showed intratumor heterogeneity of each patient. Among the tumor types, there were inter-tumor type differences in the distribution of nJSD (Fig. 4A). Notably, this inter-tumor type difference of tITH has the similar tendency to that of intratumor genetic ITH study^24^. THCA, PRAD and KIRC showed less heterogeneity than LUAD, HNSC, BLCA and LUSC (Supplementary Fig. 4). Next, we compared gITH results with our tITH result and found a positive correlation between genetic heterogeneity and tITH (Fig. 4B).

When we analyzed the cancer types separately, 4 cancer types showed similar patterns with the pan-cancer result (p-value < 0.05), HNSC (162 patients, *r* = 0.20), KIRC (64 patients, *r* = 0.27), LUAD (76 patients, *r* = 0.28) and LUSC (84 patients, *r* = 0.35), but other 3 cancer types had no relationship (p-value > 0.05), BLCA (111 patients, *r* = 0.13), PRAD (93 patients, *r* = 0.14), THCA (60 patients, *r* = −0.04). Pan-cancer trends were similar between tITH and gITH. However, there were some cancer types has weak correlation between tITH and gITH. This might due to the effect of cancer microenvironments, inter-tumor type difference and also small size of data set. In order to make sure that the pan-cancer result is not dependent on specific topology of PIN, we performed analysis using other PINs such as BioPlex and HINT^57^,^58^. Analysis of inter-PIN correlation showed that tITH was not dependent on a specific PIN topology (Supplementary Fig. 5).

In all 12 cancer data sets, the greater the number of subclones, the greater tITH values. Thus we were curious to know whether this correlation is a global trends in many different pathways in cellular mechanism. Using pathway-tITH, an average of nJSD values of genes in a pathway, we identified the pathways that pathway-tITH was correlated gITH. Most of the pathways, 255 out of 291, were significant in terms of representing ITH at the p-value < 0.001 (Pearson’s correlation test). Among the pathways identified by the pathway-tITH analysis, cell cycle and central dogma related pathways were highly correlated with the number of clones obtained from genomic ITH inference (Supplementary Table 1). This is consistent with the previous reports using genomic information that focused on variations of driver genes in cell cycle and central dogma related pathways^22^,^59^. Additionally, histological study reported cell cycle marker is correlated with gITH^24^.

### Relationship between nJSD and tumor purity

Gene expression data was used to estimate tumor purity and immune cell infiltrations^60^,^61^,^62^. Tumor purity is a measurement of sample contamination from other cell types, and immune cell infiltration is a score of immune cell proportion in a tumor sample. For tumor purity information, we used three scores– stromal score, immune score, and tumor purity score– by ESTIMATE in 1,557 patients across 8 different cancer types^60^.

We compared the tITH of each patient with the stromal score, immune score and the tumor purity score of the above mentioned methods. The stromal score was negatively associated with tITH (*r* = −0.502, p-value < 2.2e-16, Pearson’s correlation test). The immune score had a weak negative association (*r* = −0.203, p-value = 5.08e-16). The increasing proportion of specific cell types, such as stromal cells and immune cells, led to decreased tITH as expected. The tumor purity score was associated with tITH (*r* = 0.288, p-value < 2.2e-16), which is intuitive that the purer tumor will contain the more diverse clones. When analyzing cancer types separately, stromal scores had consistently negative correlation with tITH (Table 1). Immune score had negative association with tITH, and this result was consistent with the findings in a previous study^25^. Only the kidney cancer showed different patterns in tumor purity and immune score. This inverse pattern with other cancer types might be due to the higher immune cytolytic activity in kidney cancer^63^.

### Clinical potentials of tITH

Next, we were further curious if tITH has a prognostic power. We used the pan-cancer data comes with clinical information such as patient survival. We investigated the clinical utility of tITH in three different ways: survival analysis at the whole cancer cell level, pathway level, and analysis at the effect of immune cell filtration perspective.

**Figure 5:**
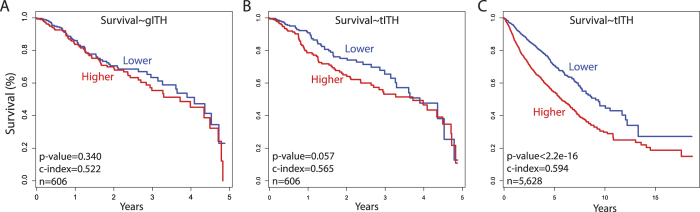
Pan-cancer survival analysis of gITH and tITH. We divided patients into two groups with median of gITH and tITH value. (**A**,**B**) was analyzed with same patients group who had reported number of subclones from other research. (**A**) Kaplan-Meier plot of the two groups based on the subclone number in 5-year censored data, and (**B**) based on tITH in 5-year censored data. (**C**) is Kaplan-Meier plot of pan-cancer patients in 12 different cancer types.

The first experiment was to investigate the prognostic power of tITH at whole cancer level (n = 606) using the Cox regression model, in comparison to that of the gITH information predicted by EXPAND. To test the prognostic power of gITH and tITH for each cancer type, we built two univariate Cox models. One was done with gITH and 5-year survival information. The other was done with tITH and 5-year survival information. The univariate Cox model using gITH was not significant (p-value = 0.3370, c-index = 0.543) while the model using tITH was statistically significant (p-value = 0.0006, c-index = 0.604). It is reported that the mutation based subclone number has a nonlinear association with survival^24^, which supports why the gITH model was not successful in separating patients groups into good or poor groups in Kaplan-Meier survival analysis. However, tITH has linear association with survival and tITH model was successful in separating patients groups (Fig. 5A,B). Next, we performed cox proportional hazard test with a bigger data set including patients without subclone information, a total of 5,628 patients. Pan-cancer univariate cox model using tITH values found clinical utility with significant statistics (c-index = 0.64, p < 2e-16). Kaplan-Meier survival analysis with poor and good group also shows significant separating of two groups (Fig. 5C).

The second experiment was to identify pathways that are relevant to or useful for the patient survival prediction. We analyzed pathway-tITH values measured for each pathway and cox proportional hazard test were done for each of the KEGG pathways. If cox model for a pathway was significant in terms of p-value < 0.001, then the pathway was selected as one that is significant for prognosis. A list of pathways that may have prognostic power is listed (Supplementary Table 2). In particular, mRNA surveillance pathway that controls mRNA abundance has the greatest c-index of 0.63. Additionally, Ribosome biogenesis in eukaryotes, Olfactory transduction, and RNA transport pathways also showed good prognostic power.

The third experiment was performed using two scores–immune score, and tumor purity score–from ESTIMATE result (n = 1,558). Previous results showed a negative correlation between tITH and immune score (Table 1). Because, recently prognostic importance of immune related cells was reported, we wanted to test that does immune score has dominant effect on clinical utility of tITH^64^. Following the gITH study reported independency of gITH from immune cell infiltration, we reproduced independency of tITH excluding effect of the immune cell infiltration^25^. We performed two variable cox proportional hazard test with both the immune score and tITH. The immune score showed weak significance (p-value = 0.52101), while tITH had significance statistics (p-value = 0.00018). Therefore, it seems that the prognostic power is more likely from heterogeneity of tumor, rather than dominant effect of immune cell proportion. When a tumor purity score from ESTIMATE was applied as covariate with tITH, cox model was improved (c-index of tITH univariate cox model: 0.568 → tITH + purity score: 0.604).

### tITH detected clonal evolution in xenograft model

We have shown that tITH can effectively measure the tumor heterogeneity from cohort data. Now we investigate whether tITH can measure the clonal evolution during tumor progression. Thus, we analyzed a time-series data of xenografted tumor by tITH in the extended time scale of tumor growth and divergence of subclones.

**Figure 6:**
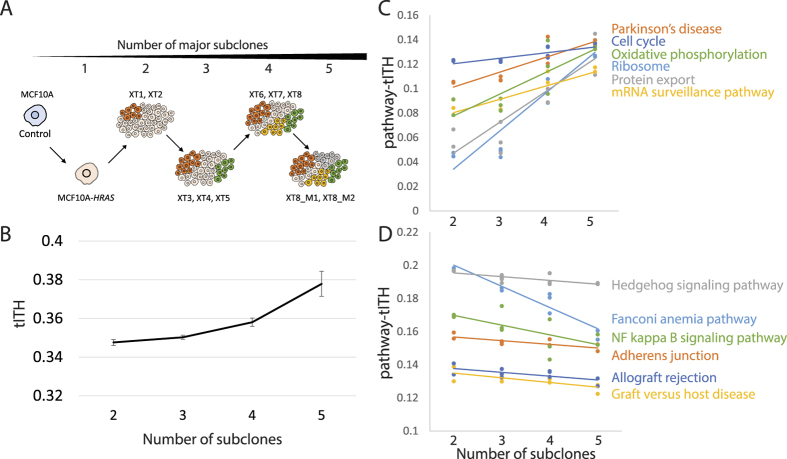
tITH during tumor evolution. (**A**) Original experimental design of the data. Single cancer cell makes 5 different subclones. (**B**) As diverging subclones, the tITH is increasing. (**C**) is pathway-tITH of 6 positively correlated KEGG pathway. This pathway getting promiscuous as diverging subclones. (**D**) is pathway-tITH of 6 negatively correlated KEGG pathways. Those pathways are converging to certain perturbed status during tumor progression and evolution.

During the tumor progression from single-cell clone to metastatic tumor, the bulk-tumor sequencing data (12 time points) revealed the emergence of different clonal populations^65^. We measured tITH of 11 time point data with first time point data (MCF10A), MCF10A-*HRAS*, XT1-XT8, M1 and M2, and compared with reported number of clones from original research (Fig. 6A).

The perturbed network status of MCF10A-*HRAS* was closer to the network of MCF10A than the xenografted tumor samples (XT1-XT8, M1 and M2). However, we observed the abrupt elevation of tITH between MCF10A-*HRAS* and XT1 (from 0.109 to 0.346). The environmental change from the culture plate to the mouse system was the first big evolutional force which might result in the dramatic network perturbation change^66^. After the xenograft, the clones continuously produced different lineages and evolved. The authors of the original research reported that the very first clone generated 5 major subclonal populations, based on mutational lineage analysis. We were able to observe the increase in tITH during that clonal divergence. The divergence of major clones occurred at MCF10A-*HRAS* → XT1, XT2 → XT3, XT5 → XT6, and XT8 → M. tITH values steadily increased as the number of subclones increased (Fig. 6B). tITH increased at MCF10A-*HRAS* → XT1 (from 0.109 to 0.346), XT2 → XT3 (from 0.349 to 0.350), XT5 → XT6 (from 0.349 to 0.356) and XT8 → M (from 0.358 to 0.384 at M1, and 0.371 at M2).

Again, we performed the pathway-tITH analysis to further explore the relationship between clonal diversity and tITH. The correlation analysis showed that a number of pathways were either positively or negatively correlated with the number of subclones. Among those pathways, metabolic pathways were highly ranked in the correlation analysis. The metabolic pathway is well known to be important in cancer mechanism^67^,^68^. For example, Oxidative phosphorylation is involved in metabolic reprograming in cancer cells^69^ and its heterogeneity is also observed clear in our correlation analysis (Fig. 6C). The whole pathway list is in Supplementary Table 3. As there are many kinds of metabolic pathways related to cancer, we focus on other pathways, excluding metabolic pathways for further pathway-tITH analysis.

We found that, in 131 out of 291 pathways, pathway-tITH values were positively correlated with tumor progression over time (*r* > 0.3, from XT1 to XT8-M2). Cell cycle and central dogma related pathways such as Parkinson’s disease, Ribosome, mRNA surveillance pathway, Cell cycle, and DNA replication were at the top of the positively correlated pathway list in *HRAS* mutated cell lines (Fig. 6C). This finding was confirmed in a study that reports the central dogma and cell cycle related pathways became more heterogeneous as the mutated *HRAS* activated MAP kinase cascades downstream and effects on transcriptional control and cell growth^70^. This result implies that ITH is highly related with the aberration in the flow of cellular information and cell cycle transition from the quiescent stem-cell like state to accelerated proliferative state. Like Cell cycle pathway, Parkinson’s disease pathway became more heterogeneous as clones diverged. Although the pathway is a kind of brain disease, it contained many genes related to cell cycle^71^. Recent cohort study revealed the relationship between parkinson’s disease and cancer^72^. Also in molecular-level studies, the *PARK2* and *LRRK2* genes well known in parkinson’s disease were revealed that those genes were related with cell cycle pathways^73^,^74^.

There were 60 of 291 pathways negatively correlated with tumor evolution measured by pathway-tITH (*r* < −0.3). The tITH values of these pathways were steady or decreased as the number of subclones increased (Fig. 6D). Thus, we conjecture that these pathways were not affected from ITH but the perturbation at the early stage of carcinogenesis remained and converged among different clones. Pathway-tITH of six pathways had a negative association with the number of subclones, especially Fanconi anemia pathway showed dramatic decrease (Fig. 6D). This indicates that Fanconi anemia pathway related to DNA repair system was heterogeneous in early time points but converged for some reasons such as the influence of the host system or the process of clonal evolution (Fig. 6D). In the original study, missense mutations on *RAD54B* and *PMS1* were reported, and they were connected by direct edges to Fanconni anemia pathway in STRING PIN (*PMS1*-*FAN1* and *RAD51*-*RAD54B*)^65^. This pathway is a genetic disease about DNA repair genes–*BRCA1, RAD51, PMS2* and FANC proteins–which are higly related to cancers. Like Fanconi anemia pathway, some pathways, such as Hedgehog signaling pathway, NF kappa B signaling pathway, Adherens junction and immune related pathways, were converged to a certain state of cancer during tumor growth (*r* < −0.6 from XT1 to XT8-M2).

## Discussion

Cancer evolution has become an important issue in understanding cancer biological mechanisms. An cancer evolution study by He *et al*.^53^, using JSD as a distance measure, reported that embryonic stem cell is the destination in cancer evolution. Their finding is that unicellularity is key characteristic of cancer^75^,^76^,^77^. Accordingly, the relationship between ESC and cancer was well studied, but the clinical application is yet to be a reality^78^,^79^. In this respect, Teschendorff group showed a possibility of clinical application in terms of cancer evolution^49^. Their study focused on signaling pathways, or regimes in their term, and observed the reverse differentiation of cancer cells^48^. The study used a network perturbation concept in terms of entropy and reported the association between differentiation potential and network entropy, thus they defined the measure termed as Signaling entropy. This way, the study showed the clinical importance of the signaling entropy in cancer, and reported the relationship with ITH^49^. The study discussed about a possibility of measuring ITH using the network entropy.

However, Signaling entropy was difficult to distinguish differentiation potential from ITH because the network measure is the metric of entire PIN. On the contrary, our entropy measure is gene centric and then combines gene level information to pathway level and also to the entire PIN level. We used the metric to measure ITH in terms of subclone diversity, rather than focusing on the differential potential.

Our analysis results in Fig. 4B with the tITH approach in the xenograft tumor evolution data can be interpreted as an increment of differentiation potential in terms of the reverse-evolution hypothesis of the previous works. To investigate further, we analyzed heterogeneous data by incrementally adding transcriptome data form different cancer cell lines (Fig. 3). Differentiation potentials in different cell lines may not be significantly different, thus the increased network entropy observed in our study with the mixed cell line data may be from other factors, possibly from some pathways, rather than differentiation related pathways. This hypothesis was supported by the analysis of the clonal evolution data. tITH values of differentiation potential related pathways were either steady or slightly decreased (Fig. 6D). This implies that cancer clones lose their control of differentiation at the early carcinogenesis stage. After tumorigenesis, continuous increment in tITH values may be the result from heterogeneity in cell cycle and central dogma related pathways. This conjecture is supported by the previous work with histological information^24^.

Although our study highlights the importance of cell cycle pathways in terms of tumor heterogeneity, reverse differentiation is well documented and important factor in cancer evolution^77^. In cellular mechanism, multiple signaling pathways work as control switch between differentiation and proliferation state^80^. In our analysis, tITH value of the Hedgehog signaling pathway, an important information transmitor during embryogenesis^81^, remains unchanged for different number of subclones, while cell cycle related pathways become more ambiguous. However, it is still unclear how the loss of differentiation and the accelerated cell proliferation interplay. Clones may have loss of differentiation because of the fast and uncotrolled cell cycle, however simultaneously cancer stem cell population did not differentiate even they were quiescent state of cell cycle^82^,^83^. The aberration of the master regulator of differentiation and proliferation - like hedgehog signaling that we found - may be the main cause of dysregulation of differentiation. A breakthrough in cancer may be in there^76^.

Our method successfully measured ITH with transcriptome data and network information, but our method did not use whole features of transcriptome data. Biological network information includes only a small number of genes; 20,000~40,000 transcripts are generally observed in the whole transcriptome data but only ~10,000 genes are used in the interaction data. There could be another approaches with *de novo* network construction with edge probability using statistical approaches^84^,^85^. As more comprehensive interaction data, including regulatory data, is available, our method can be more accurate in predicting ITH.

Although our study was able to show tumor heterogeneity using four datasets, our computational methods still need to be improved. Our approach focused on detecting ITH with bulk RNA-sequencing data. There are other notable methods which deconvolute gene-expression data to identify population of specific cell types^86^. Especially, immune related cell population in tumor sample were well studied^31^,^64^. These methods, although successful in de-composing cell populations, require a reference gene expression profile. The requirement for a reference gene expression profile makes difficult to measure ITH since the number of clones is not known; this is a typical chicken and egg problem. Our current method is to measure the heterogeneity in a systematic view but it is not designed to de-compose cancer clones. As a future study, we are working on a computational method that can both de-compose clones and measure the status of heterogeneity.

We propose a new approach, tITH, to inference ITH using RNA-seq data by nJSD and compared with gITH. Our tITH was in agreement with gITH. Since our method is to measure the status of gene expression, it is possible to perform functional or pathway-level analysis. With xenograft model, we found importance of cell cycle related pathways in ITH. Other signaling pathway showed converging tendency during clonal evolutions. In addition, we showed that tITH achieved better performance than gITH in cox regression model analysis for survival prediction. We believe that ITH should be investigated at the full spectrum of the central dogma, *i.e*., at DNA, RNA, and protein levels. Our tITH can be useful for ITH inference using RNA-sequencing data of the bulk tumor, which may be useful for developing cost effective molecular diagnosis methods.

## Materials

### Gene Expression Data

We used four different datasets to examine the usefulness of nJSD. The lung adenocarcinoma (LUAD) single-cell RNA-sequencing data from GEO under the accession number of GSE69405^54^. Human cancer cell lines data, 675 different human origin cell lines was obtained from GEO under the accession number of GSE30611^87^. The xenograft-tumor data was obtained from GSE63630. The Ensembl gene ID was converted to gene symbol using *mygene* 2.3.0 python package. The log transformed gene expression data was downloaded from the supplementary data of the published research^65^. The pan-cancer data, TCGA RNA Seq V2, was obtained from TCGA data portal (https://tcga-data.nci.nih.gov/tcga/). We selected cancer types which have more than 10 normal samples: Bladder Urothelial Carcinoma (BLCA), Breast invasive carcinoma (BRCA), Colon adenocarcinoma (COAD), Head and Neck squamous cell carcinoma (HNSC), Kidney renal clear cell carcinoma (KIRC), Kidney renal papillary cell carcinoma (KIRP), Lung adenocarcinoma (LUAD), Lung squamous cell carcinoma (LUSC), Prostate adenocarcinoma (PRAD), Thyroid carcinoma (THCA) and Uterine Corpus Endometrial Carcinoma (UCEC).

### Pathway and Protein Interaction network

Protein-protein interaction network was constructed with STRING v9 data^88^. 479,635 edges and 10,100 genes consist PIN. BioPlex and HINT were used in inter-PIN comparison study^57^,^58^. BioPlex network has 10,963 genes and 56,554 edges and HINT has 12,194 genes and 53,126 edges. The KEGG pathway data contained 295 pathways and 6,969 genes.

### *In silico* simulation with 675 human cancer cell lines data

We created *in silico* heterogeneous tumor data with gene-expression data of 675 human cancer cell lines. Randomly selected 2, 4, 8, 16, 32, 64, 128, 256, and 512 out of 675 cell line gene-expression data was individually averaged into a single gene-expression profile. Each simulation data had 1,000 gene-expression profiles.

### tITH calculation of TCGA patients and comparison to gITH results

We calculated tITH and pathway-tITH based on mean expression level of multiple normal samples of each cancer type. The intratumor heterogeneity, number of clones, in TCGA patients data were obtained from a previously published research^24^. This intratumor heterogeneity information was calculated based on mutations using state-of-the-art tools, PyClone and EXPANDs. The tumor purity information of TCGA patients is obtained from a previously published research^60^. This tool for tumor purity estimation, ESTIMATE, produces score about immune cell infiltration, stromal cell population and tumor purity. The TCGA pan-cancer clinical data were downloaded from TCGA data portal. Cox regression model analysis was done by using R library survival^89^.

## Additional Information

**How to cite this article**: Park, Y. *et al*. Measuring intratumor heterogeneity by network entropy using RNA-seq data. *Sci. Rep.* **6**, 37767; doi: 10.1038/srep37767 (2016).

**Publisher’s note:** Springer Nature remains neutral with regard to jurisdictional claims in published maps and institutional affiliations.

## Supplementary Material

Supplementary Information

## Figures and Tables

**Figure 1:**
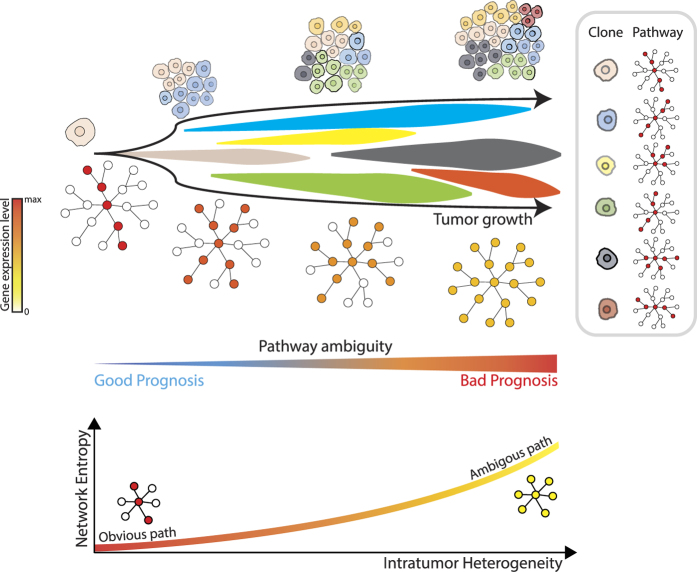
Pathway ambiguity model to analyze transcriptome-based ITH. The figure illustrates the heterogeneous tumor and its corresponding pathway status. While the clonal evolution produces different subclonal populations, pathway is getting ambiguous. Here, different clones are associated with their differentially activated pathway. In this context, measuring network perturbation by network entropy implies measuring pathway ambiguity. As ITH getting worse, the entropy of network increases.

**Table 1:** Relationship between tITH and tumor purity score from ESTIMATE.

	BLCA	BRCA	COAD	HNSC	LUAD	LUSC	KIRC
Patients (n)	95	471	18	291	228	129	326
Purity (*r*)	0.330	0.417	0.466	0.459	0.230	0.505	0.074
Immune Score (*r*)	−0.389	−0.184	−0.481	−0.251	−0.382	−0.487	0.177
Stromal Score (*r*)	−0.575	−0.585	−0.616	−0.515	−0.384	−0.476	−0.342

Individual cancer type comparison of Pearson’s correlation coefficients.
