# Supplementary material for: Measuring intratumor heterogeneity by network entropy using RNA-seq data
Source: Sci Rep. 2016 Nov 24;6:37767. doi: 10.1038/srep37767 (PMC5121893; doi:10.1038/srep37767)
Supplement: Supplementary Information [file srep37767-s1.pdf]

Supplementary Figures

Measuring intratumor heterogeneity by network entropy using RNA-seq data

Youngjune Park, Sangsoo Lim, Jin-Wu Nam and Sun Kim

(a)

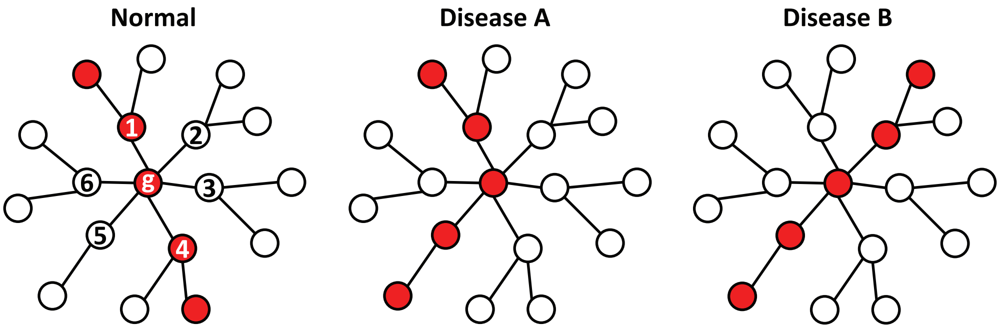

(b)

	Normal	Disease A	Disease B
**Gene Expression of neighbor of "g"**	{ 4, 1, 1, 4, 1, 1 }	{ 4, 1, 1, 1, 4, 1 }	{ 1, 4, 1, 1, 4, 1 }
**Probability Distribution of "g"**	$\{\frac{4}{12},\frac{1}{12},\frac{1}{12},\frac{4}{12},\frac{1}{12},\frac{1}{12}\}$	$\{\frac{4}{12},\frac{1}{12},\frac{1}{12},\frac{1}{12},\frac{4}{12},\frac{1}{12}\}$	$\{\frac{1}{12},\frac{4}{12},\frac{1}{12},\frac{1}{12},\frac{4}{12},\frac{1}{12}\}$
	**Normal - Normal**	**Normal - Disease A**	**Normal - Disease B**
**JSD of "g"**	0.0	0.08	0.16
**Average JSD of all genes (nJSD)**	0.0	0.010	0.020

Figure 1: **nJSD calculation example** A network consist of 18 genes and 17 edges. (a) All 3 networks have the same gene expression level in total, but the activated paths are different. (b) An example of calculating JSD of the gene "g". nJSD of each network is calculated based on normal network. With gene expression of neighbors, interacting probability is defined and JSD is calculated. Mean JSD of all genes in network is nJSD. Detailed information can be found in the Method section.

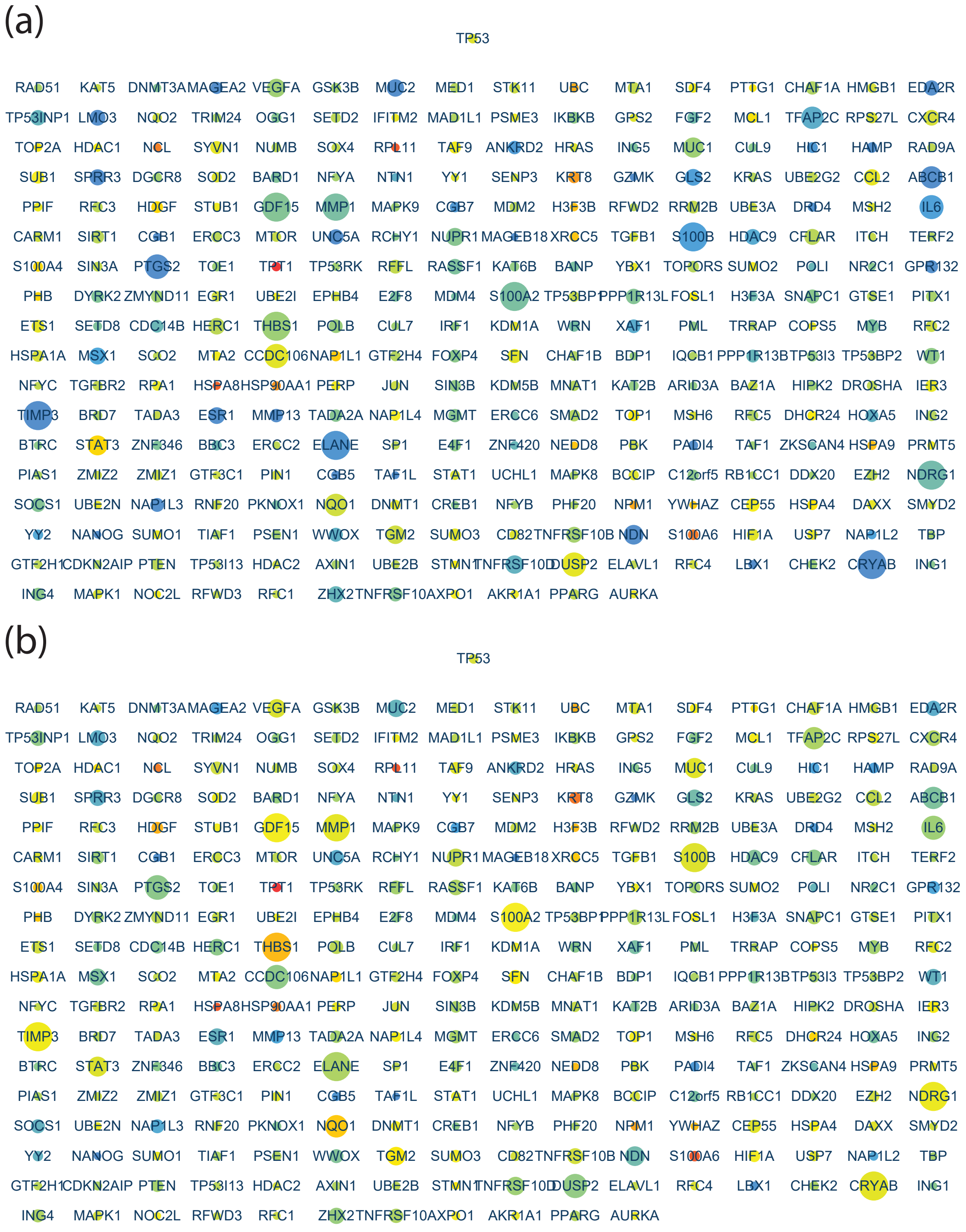

Figure 2: **Protein-interaction network of *TP53* gene and its neighbors of *in silico* mixed data, X2 and X256.** (a) is for x2 mixed data. (b) is for x256 mixed data. The size of circle is representing gene-expression difference between two data.

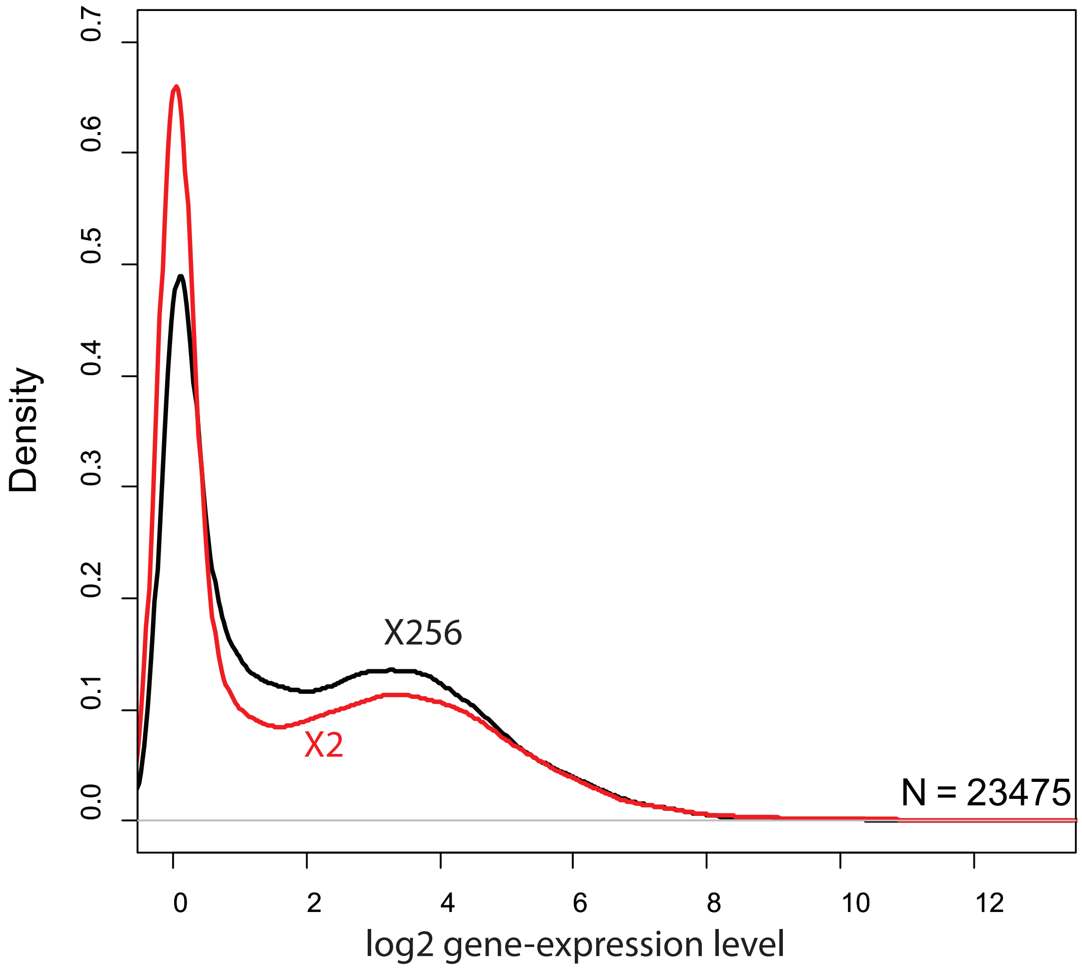

Figure 3: **Gene expression distribution of simulated data.** X2 represents mixed data of couple cancer cell line data. X256 represents mixed data of 256 different cancer cell line data.

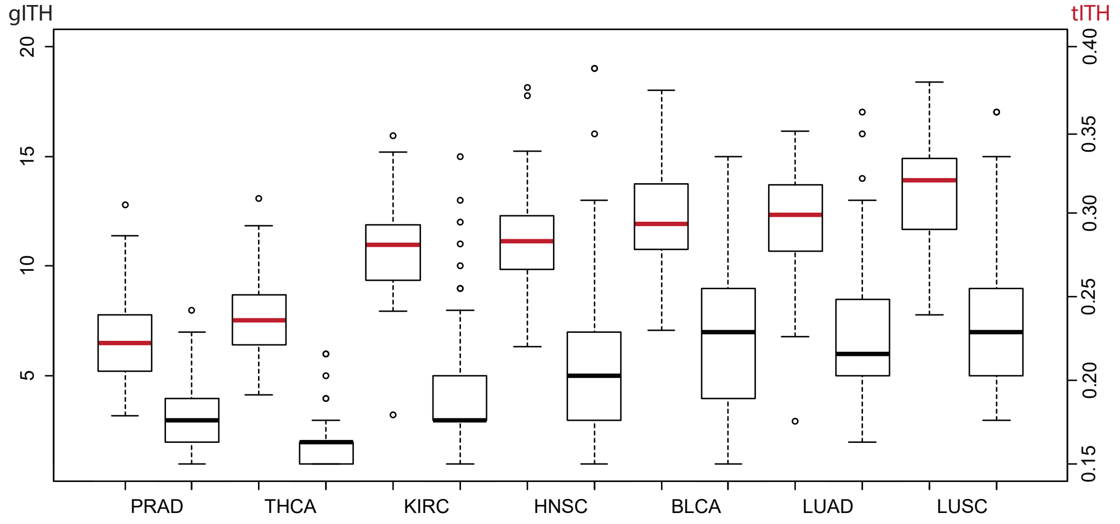

Figure 4: **Comparison gITH and tITH in cancer type seperatly** The boxes with red line is about tITH and the boxes with black line is about gITH. In seven different cancer types, the trends between gITH and tITH is similar. This results is obtained from TCGA 648 patients.

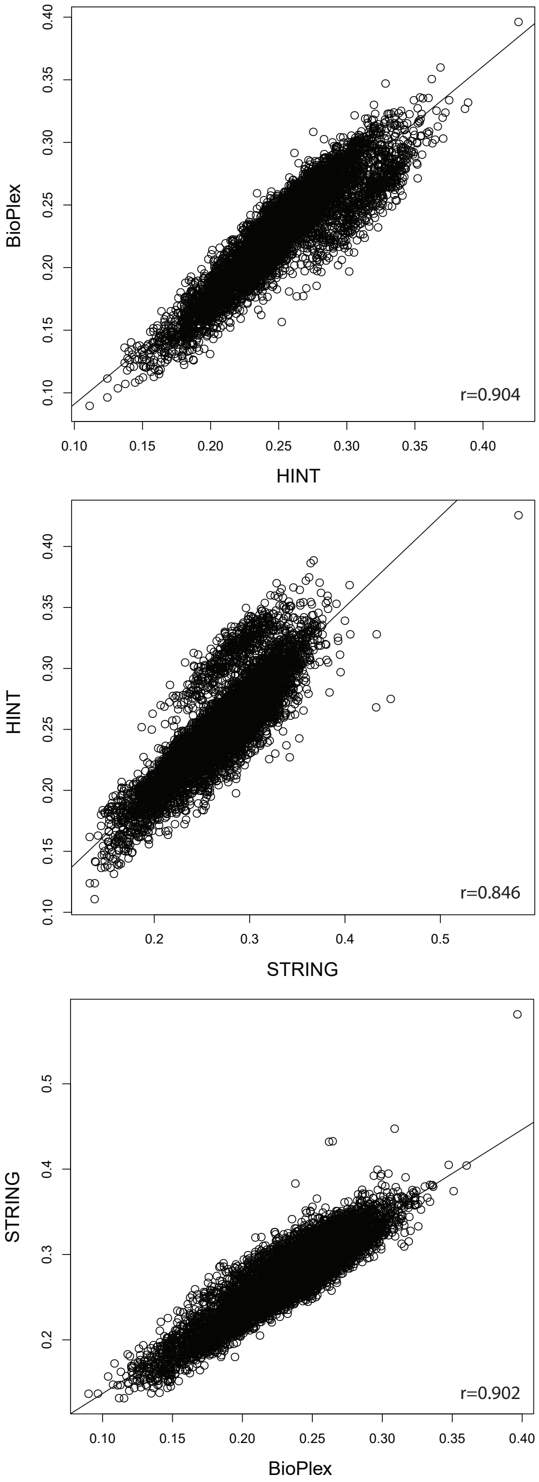

Figure 5: **Inter-PIN Correlation of tTIH** We applied 3 different PIN on tITH analysis of TCGA pan-cancer cohort (n=6320). Three PIN results are highly correlated with each others.

Supplementary Table1

Measuring intratumor heterogeneity by network entropy using RNA-seq data

Youngjune Park, Sangsoo Lim, Jin-Wu Nam and Sun Kim

Correlation between gITH and KEGG pathway-tITH

KEGG_pathway	r	p-value
Fanconi_anemia_pathway	0.543198	0
Cell_cycle	0.538414	0
p53_signaling_pathway	0.522782	0
Nucleotide_excision_repair	0.51885	0
Homologous_recombination	0.513607	0
DNA_replication	0.507205	0
Mismatch_repair	0.497014	0
Base_excision_repair	0.487229	0
Ubiquitin_mediated_proteolysis	0.479705	0
Oocyte_meiosis	0.474504	0
Spliceosome	0.47327	0
Olfactory_transduction	0.471522	0
Proteasome	0.469864	0
MicroRNAs_in_cancer	0.468819	0
RNA_polymerase	0.465411	0
Colorectal_cancer	0.46512	0
RNA_degradation	0.461172	0
Endometrial_cancer	0.460541	0
Protein_processing_in_endoplasmic_reticulum	0.459743	0
Pancreatic_cancer	0.453766	0
RNA_transport	0.449303	0
Bladder_cancer	0.446358	0
Shigellosis	0.445264	0
Small_cell_lung_cancer	0.443958	0
Non.small_cell_lung_cancer	0.433144	0
Progesterone.mediated_oocyte_maturation	0.432866	0

Neurotrophin_signaling_pathway	0.431419	0
Bacterial_invasion_of_epithelial_cells	0.430792	0
Pathogenic_Escherichia_coli_infection	0.430407	0
Acute_myeloid_leukemia	0.425022	0
Chronic_myeloid_leukemia	0.424043	0
Central_carbon_metabolism_in_cancer	0.423329	0
Glioma	0.421634	0
Cholinergic_synapse	0.419081	0
Hepatitis_C	0.417997	0
Dopaminergic_synapse	0.417469	0
MAPK_signaling_pathway	0.415803	0
Legionellosis	0.414864	0
mRNA_surveillance_pathway	0.41447	0
Huntington.s_disease	0.414193	0
Glutamatergic_synapse	0.411933	0
Prostate_cancer	0.411479	0
Basal_transcription_factors	0.40701	0
Circadian_entrainment	0.406464	0
Thyroid_hormone_signaling_pathway	0.403801	0
AGE.RAGE_signaling_pathway_in_diabetic_complications	0.4037	0
Arrhythmogenic_right_ventricular_cardiomyopathy_.ARVC.	0.402922	0
Alcoholism	0.399739	0
Neuroactive_ligand.receptor_interaction	0.399632	0
Ras_signaling_pathway	0.399035	0
Pathways_in_cancer	0.397076	0
Insulin_secretion	0.395829	0
Signaling_pathways_regulating_pluripotency_of_stem_cells	0.394286	0
Platelet_activation	0.393453	0
Aldosterone_synthesis_and_secretion	0.391316	0
Retrograde_endocannabinoid_signaling	0.391295	0
Epstein.Barr_virus_infection	0.38948	0
Renal_cell_carcinoma	0.388641	0
ECM.receptor_interaction	0.388539	0
Influenza_A	0.38838	0
Hepatitis_B	0.384782	0
VEGF_signaling_pathway	0.384504	0
Inositol_phosphate_metabolism	0.384074	0
Rap1_signaling_pathway	0.38373	0
Toxoplasmosis	0.383228	0
GABAergic_synapse	0.382887	0
X2.Oxocarboxylic_acid_metabolism	0.381961	0
Glycosaminoglycan_degradation	0.38121	0
Cocaine_addiction	0.381063	0
Thyroid_cancer	0.380732	0

TGF.beta_signaling_pathway	0.379923	0
ErbB_signaling_pathway	0.379639	0
Leukocyte_transendothelial_migration	0.378326	0
Apoptosis	0.377509	0
HIF.1_signaling_pathway	0.377479	0
Salivary_secretion	0.376542	0
mTOR_signaling_pathway	0.375862	0
Amyotrophic_lateral_sclerosis_.ALS.	0.375781	0
Amphetamine_addiction	0.375413	0
Phospholipase_D_signaling_pathway	0.374733	0
PI3K.Akt_signaling_pathway	0.3747	0
Parkinson.s_disease	0.373681	0
cAMP_signaling_pathway	0.372912	0
Galactose_metabolism	0.372654	0
Long.term_potentiation	0.372223	0
Calcium_signaling_pathway	0.371914	0
Transcriptional_misregulation_in_cancer	0.371452	0
GnRH_signaling_pathway	0.370108	0
Adrenergic_signaling_in_cardiomyocytes	0.369759	0
Oxytocin_signaling_pathway	0.367641	0
HTLV.I_infection	0.367472	0
NOD.like_receptor_signaling_pathway	0.366893	0
Herpes_simplex_infection	0.366749	0
Viral_carcinogenesis	0.365942	0
Thyroid_hormone_synthesis	0.364638	0
Pertussis	0.363626	0
Focal_adhesion	0.360435	0
Alzheimer.s_disease	0.359795	0
Regulation_of_lipolysis_in_adipocytes	0.359118	0
Aldosterone.regulated_sodium_reabsorption	0.358607	0
Ovarian_steroidogenesis	0.358517	0
TNF_signaling_pathway	0.357906	0
Morphine_addiction	0.357752	0
Fc_epsilon_RI_signaling_pathway	0.357669	0
Sphingolipid_signaling_pathway	0.35719	0
Chagas_disease_.American_trypanosomiasis.	0.355683	0
T_cell_receptor_signaling_pathway	0.354586	0
Nicotine_addiction	0.353161	0
Ribosome_biogenesis_in_eukaryotes	0.352439	0
Proteoglycans_in_cancer	0.351403	0
Prion_diseases	0.35064	0
Circadian_rhythm	0.349259	0
Pancreatic_secretion	0.347105	0
Leishmaniasis	0.34584	0

Axon_guidance	0.343406	0
Biosynthesis_of_unsaturated_fatty_acids	0.343036	0
Serotonergic_synapse	0.342571	0
cGMP.PKG_signaling_pathway	0.341802	0
Gastric_acid_secretion	0.341441	0
Prolactin_signaling_pathway	0.339914	0
Type_II_diabetes_mellitus	0.339712	0
Sulfur_metabolism	0.339337	0
Adherens_junction	0.3385	0
Long.term_depression	0.336863	0
Fatty_acid_metabolism	0.33675	0
Complement_and_coagulation_cascades	0.336148	0
Gap_junction	0.334532	0
Notch_signaling_pathway	0.334525	0
Vascular_smooth_muscle_contraction	0.334482	0
Inflammatory_bowel_disease_.IBD.	0.333411	0
Cell_adhesion_molecules_.CAMs.	0.332478	0
Fc_gamma_R.mediated_phagocytosis	0.331579	0
AMPK_signaling_pathway	0.330607	0
Inflammatory_mediator_regulation_of_TRP_channels	0.330465	0
Melanoma	0.32983	0
Renin_secretion	0.329241	0
Osteoclast_differentiation	0.327064	0
Hippo_signaling_pathway	0.327003	0
Estrogen_signaling_pathway	0.322809	0
Viral_myocarditis	0.321909	0
Biotin_metabolism	0.321717	0
Bile_secretion	0.320649	0
Salmonella_infection	0.319155	0
Taste_transduction	0.318834	0
Measles	0.315029	2.22E-16
Regulation_of_actin_cytoskeleton	0.314968	2.22E-16
Endocytosis	0.314288	2.22E-16
Steroid_hormone_biosynthesis	0.314118	2.22E-16
Endocrine_and_other_factor.regulated_calcium_reabsorption	0.313207	4.44E-16
Tuberculosis	0.312944	4.44E-16
Amoebiasis	0.310864	4.44E-16
Vasopressin.regulated_water_reabsorption	0.310822	4.44E-16
Antigen_processing_and_presentation	0.30821	8.88E-16
African_trypanosomiasis	0.306446	1.33E-15
Systemic_lupus_erythematosus	0.304492	2.22E-15
Linoleic_acid_metabolism	0.303653	2.44E-15
Chemokine_signaling_pathway	0.303244	2.66E-15
Dilated_cardiomyopathy	0.303052	2.89E-15

Carbon_metabolism	0.299682	5.77E-15
Cytokine.cytokine_receptor_interaction	0.293459	2.24E-14
NF.kappa_B_signaling_pathway	0.293448	2.24E-14
FoxO_signaling_pathway	0.292734	2.62E-14
Hypertrophic_cardiomyopathy_.HCM.	0.292325	2.84E-14
Carbohydrate_digestion_and_absorption	0.291261	3.55E-14
Phosphatidylinositol_signaling_system	0.290749	3.95E-14
Autoimmune_thyroid_disease	0.290538	4.13E-14
Toll.like_receptor_signaling_pathway	0.288183	6.75E-14
Basal_cell_carcinoma	0.28805	6.93E-14
Non.alcoholic_fatty_liver_disease_.NAFLD.	0.287008	8.62E-14
Melanogenesis	0.286022	1.05E-13
Metabolic_pathways	0.28602	1.05E-13
Jak.STAT_signaling_pathway	0.285684	1.13E-13
Maturity_onset_diabetes_of_the_young	0.283624	1.72E-13
N.Glycan_biosynthesis	0.28282	2.03E-13
Arachidonic_acid_metabolism	0.278818	4.53E-13
Ubiquinone_and_other_terpenoid.quinone_biosynthesis	0.277541	5.84E-13
Malaria	0.277534	5.84E-13
Adipocytokine_signaling_pathway	0.2775	5.88E-13
Protein_digestion_and_absorption	0.277412	5.99E-13
Thiamine_metabolism	0.275254	9.16E-13
Fatty_acid_elongation	0.273499	1.29E-12
Tyrosine_metabolism	0.271899	1.76E-12
RIG.I.like_receptor_signaling_pathway	0.270115	2.49E-12
Staphylococcus_aureus_infection	0.26735	4.22E-12
Phagosome	0.267153	4.38E-12
Purine_metabolism	0.265737	5.73E-12
Fructose_and_mannose_metabolism	0.264932	6.67E-12
Asthma	0.264037	7.90E-12
Lysosome	0.262264	1.10E-11
Butanoate_metabolism	0.261433	1.28E-11
Propanoate_metabolism	0.260922	1.41E-11
Insulin_signaling_pathway	0.260768	1.45E-11
Wnt_signaling_pathway	0.260354	1.57E-11
Sulfur_relay_system	0.259979	1.68E-11
Choline_metabolism_in_cancer	0.259628	1.79E-11
Phototransduction	0.259618	1.79E-11
Allograft_rejection	0.257439	2.67E-11
Cardiac_muscle_contraction	0.256867	2.97E-11
Hematopoietic_cell_lineage	0.256734	3.04E-11
B_cell_receptor_signaling_pathway	0.253968	5.01E-11
Glucagon_signaling_pathway	0.251352	8.00E-11
Drug_metabolism	0.250011	1.01E-10

Pyrimidine_metabolism	0.2439	2.94E-10
Natural_killer_cell_mediated_cytotoxicity	0.242677	3.63E-10
Graft.versus.host_disease	0.241678	4.31E-10
One_carbon_pool_by_folate	0.239588	6.14E-10
Proximal_tubule_bicarbonate_reclamation	0.236248	1.07E-09
Insulin_resistance	0.230231	2.88E-09
Phenylalanine_metabolism	0.227338	4.58E-09
SNARE_interactions_in_vesicular_transport	0.22693	4.89E-09
Type_I_diabetes_mellitus	0.223122	8.91E-09
Glyoxylate_and_dicarboxylate_metabolism	0.22189	1.08E-08
Tight_junction	0.218739	1.76E-08
Valine._leucine_and_isoleucine_degradation	0.214694	3.24E-08
Pentose_phosphate_pathway	0.213677	3.78E-08
Ribosome	0.211416	5.29E-08
Selenocompound_metabolism	0.208628	7.96E-08
Cysteine_and_methionine_metabolism	0.20447	1.45E-07
Glycerophospholipid_metabolism	0.203322	1.71E-07
Fatty_acid_biosynthesis	0.202231	2.00E-07
Ether_lipid_metabolism	0.201413	2.24E-07
Hedgehog_signaling_pathway	0.199123	3.08E-07
Peroxisome	0.199088	3.10E-07
Rheumatoid_arthritis	0.192012	8.14E-07
Tryptophan_metabolism	0.189435	1.15E-06
Metabolism_of_xenobiotics_by_cytochrome_P450	0.186727	1.64E-06
Biosynthesis_of_amino_acids	0.186594	1.67E-06
alpha.Linolenic_acid_metabolism	0.183863	2.37E-06
Arginine_and_proline_metabolism	0.183429	2.51E-06
Cytosolic_DNA.sensing_pathway	0.183267	2.56E-06
Sphingolipid_metabolism	0.183014	2.64E-06
Starch_and_sucrose_metabolism	0.176766	5.79E-06
Other_glycan_degradation	0.172609	9.62E-06
Intestinal_immune_network_for_IgA_production	0.171943	1.04E-05
Glutathione_metabolism	0.170791	1.20E-05
Lysine_degradation	0.169491	1.40E-05
Taurine_and_hypotaurine_metabolism	0.168238	1.62E-05
Non.homologous_end.joining	0.167119	1.85E-05
Renin.angiotensin_system	0.163524	2.80E-05
beta.Alanine_metabolism	0.162945	2.99E-05
Dorso.ventral_axis_formation	0.162337	3.20E-05
Alanine._aspartate_and_glutamate_metabolism	0.160234	4.06E-05
Chemical_carcinogenesis	0.156403	6.21E-05
Glycosylphosphatidylinositol.GPI..anchor_biosynthesis	0.1561	6.42E-05
Synaptic_vesicle_cycle	0.151847	0.000102
Phenylalanine._tyrosine_and_tryptophan_biosynthesis	0.151712	0.000103

Primary_immunodeficiency	0.15109	0.00011
Nitrogen_metabolism	0.147334	0.000163
Folate_biosynthesis	0.146211	0.000184
Glycolysis_Gluconeogenesis	0.141239	0.000304
Glycerolipid_metabolism	0.139644	0.000356
Aminoacyl.tRNA_biosynthesis	0.138696	0.00039
ABC_transporters	0.136497	0.000484
Epithelial_cell_signaling_in_Helicobacter_pylori_infection	0.132463	0.00071
D.Glutamine_and_D.glutamate_metabolism	0.130519	0.000852
Fatty_acid_degradation	0.122399	0.00177
Other_types_of_O.glycan_biosynthesis	0.121004	0.001999
Caffeine_metabolism	0.120338	0.002117
PPAR_signaling_pathway	0.119478	0.002279
Primary_bile_acid_biosynthesis	0.115201	0.003269
Butirosin_and_neomycin_biosynthesis	0.113666	0.003711
Fat_digestion_and_absorption	0.111449	0.004444
Arginine_biosynthesis	0.106716	0.006464
Histidine_metabolism	0.10261	0.008847
Retinol_metabolism	0.09335	0.017285
Pentose_and_glucuronate_interconversions	0.093179	0.017492
Vitamin_digestion_and_absorption	0.092795	0.017963
Glycosaminoglycan_biosynthesis	0.077491	0.04829
Pyruvate_metabolism	0.069141	0.078157
Glycine._serine_and_threonine_metabolism	0.066138	0.092029
Mineral_absorption	0.065579	0.094818
Degradation_of_aromatic_compounds	0.064401	0.100913
Ascorbate_and_aldarate_metabolism	0.059404	0.130297
Porphyrin_and_chlorophyll_metabolism	0.059121	0.132141
Glycosphingolipid_biosynthesis	0.056428	0.150711
Citrate_cycle_.TCA_cycle.	0.05367	0.171727
Synthesis_and_degradation_of_ketone_bodies	0.051114	0.193084
Steroid_biosynthesis	0.049092	0.211314
Terpenoid_backbone_biosynthesis	0.041101	0.295422
Pantothenate_and_CoA_biosynthesis	0.040946	0.297245
Vitamin_B6_metabolism	0.027802	0.4792
Nicotinate_and_nicotinamide_metabolism	0.025895	0.509868
Vibrio_cholerae_infection	0.024648	0.530475
Regulation_of_autophagy	0.022497	0.566955
Oxidative_phosphorylation	0.022371	0.569135
Protein_export	0.020397	0.603703
D.Arginine_and_D.ornithine_metabolism	0.014019	0.721275
Mucin_type_O.Glycan_biosynthesis	0.008563	0.827503
Cyanoamino_acid_metabolism	-0.00888	0.821262
Riboflavin_metabolism	-0.04637	0.237758

Collecting_duct_acid_secretion	-0.04848	0.217057
Amino_sugar_and_nucleotide_sugar_metabolism	-0.15592	6.55E-05
Lipoic_acid_metabolism	NA	1
Valine._leucine_and_isoleucine_biosynthesis	NA	1
Lysine_biosynthesis	NA	1

Supplementary Table2

Measuring intratumor heterogeneity by network entropy using RNA-seq data

Youngjune Park, Sangsoo Lim, Jin-Wu Nam and Sun Kim

TCGA pan-cancer cox model analysis with pathway-tITH

KEGG pathway	p-value	c-index
mRNA_surveillance_pathway	0	0.630359
Ribosome_biogenesis_in_eukaryotes	0	0.625562
Olfactory_transduction	0	0.617829
RNA_transport	0	0.610876
Purine_metabolism	0	0.604419
Influenza_A	2.66E-13	0.598796
Basal_cell_carcinoma	2.12E-12	0.594936
Homologous_recombination	0	0.592557
Cell_cycle	1.36E-13	0.590326
Hepatitis_C	5.83E-10	0.589985
Carbon_metabolism	1.96E-11	0.58984
Nucleotide_excision_repair	1.07E-14	0.589662
Fanconi_anemia_pathway	2.89E-15	0.589434
Fatty_acid_elongation	0	0.589344
p53_signaling_pathway	8.61E-11	0.5889
Endometrial_cancer	4.22E-08	0.587383
Glycosylphosphatidylinositol(GPI)-anchor_biosynthesis	6.71E-13	0.586171
Colorectal_cancer	2.30E-07	0.584896
Inflammatory_bowel_disease_(IBD)	4.84E-09	0.584664
Phosphatidylinositol_signaling_system	2.05E-07	0.584458
Mismatch_repair	1.79E-14	0.583654
Pathogenic_Escherichia_coli_infection	1.82E-08	0.582843
Oocyte_meiosis	8.47E-08	0.582668
DNA_replication	2.43E-14	0.581975
Fc_epsilon_RI_signaling_pathway	1.70E-06	0.581655
Prion_diseases	4.72E-09	0.580111

Leishmaniasis	4.76E-08	0.579405
Bladder_cancer	3.14E-07	0.577553
Phototransduction	2.03E-09	0.577298
Serotonergic_synapse	1.42E-08	0.577109
Asthma	4.60E-08	0.576683
Progesterone-mediated_oocyte_maturation	3.45E-06	0.576461
HTLV-I_infection	6.83E-07	0.576229
Proteasome	7.18E-10	0.576095
Pancreatic_cancer	1.74E-06	0.575228
MicroRNAs_in_cancer	1.71E-06	0.575029
VEGF_signaling_pathway	1.54E-05	0.574417
Taste_transduction	1.51E-06	0.574338
Huntington's_disease	3.49E-07	0.574285
ABC_transporters	6.31E-06	0.573862
GABAergic_synapse	6.00E-08	0.573662
RNA_polymerase	1.00E-07	0.573635
Spliceosome	8.60E-07	0.573504
Inositol_phosphate_metabolism	2.87E-06	0.57272
Pathways_in_cancer	1.28E-05	0.572523
AMPK_signaling_pathway	4.61E-06	0.571889
Non-small_cell_lung_cancer	4.30E-06	0.571851
Sphingolipid_signaling_pathway	9.01E-05	0.571239
Neurotrophin_signaling_pathway	0.000214	0.571205
AGE-RAGE_signaling_pathway_in_diabetic_complications	1.95E-05	0.570945
Hedgehog_signaling_pathway	6.17E-06	0.570177
Thiamine_metabolism	2.52E-07	0.56998
Chagas_disease_(American_trypanosomiasis)	2.17E-05	0.569889
Prostate_cancer	1.92E-05	0.569712
Aldosterone_synthesis_and_secretion	8.54E-05	0.569512
Dopaminergic_synapse	1.32E-05	0.569255
Biosynthesis_of_unsaturated_fatty_acids	1.47E-05	0.567897
Glioma	0.000206	0.567444
Chronic_myeloid_leukemia	6.44E-05	0.567329
Osteoclast_differentiation	8.88E-05	0.567213
Retrograde_endocannabinoid_signaling	7.28E-06	0.567114
Fatty_acid_degradation	0.000218	0.566975
African_trypanosomiasis	2.84E-06	0.566963
Signaling_pathways_regulating_pluripotency_of_stem_cells	2.40E-06	0.566839
Shigellosis	0.000106	0.566431
Acute_myeloid_leukemia	9.92E-05	0.56584
beta-Alanine_metabolism	2.34E-07	0.565677
Phospholipase_D_signaling_pathway	0.00055	0.565189
Apoptosis	0.000191	0.565041
Hepatitis_B	3.53E-05	0.564969

Estrogen_signaling_pathway	2.89E-05	0.564662
Platelet_activation	0.000356	0.56462
T_cell_receptor_signaling_pathway	0.000179	0.564331
Drug_metabolism	1.52E-08	0.564085
Proteoglycans_in_cancer	7.33E-05	0.56404
PI3K-Akt_signaling_pathway	8.31E-05	0.563607
Glyoxylate_and_dicarboxylate_metabolism	1.16E-06	0.563425
Herpes_simplex_infection	7.99E-05	0.563399
Gap_junction	0.000439	0.56326
One_carbon_pool_by_folate	8.81E-06	0.563019
Wnt_signaling_pathway	7.01E-05	0.562992
Carbohydrate_digestion_and_absorption	2.02E-05	0.562814
Propanoate_metabolism	1.66E-12	0.562486
Cholinergic_synapse	3.74E-05	0.562178
Toxoplasmosis	0.000732	0.562146
Jak-STAT_signaling_pathway	0.000333	0.562139
Ubiquitin_mediated_proteolysis	3.15E-05	0.561526
Morphine_addiction	4.05E-05	0.561089
Small_cell_lung_cancer	0.000142	0.561081
Cell_adhesion_molecules_(CAMs)	9.55E-05	0.561003
Glutathione_metabolism	0.000102	0.560622
Long-term_depression	0.000157	0.560575
Hematopoietic_cell_lineage	6.53E-06	0.560484
Axon_guidance	0.000485	0.560309
Type_I_diabetes_mellitus	4.48E-06	0.560268
Hippo_signaling_pathway	7.58E-05	0.559419
Insulin_signaling_pathway	0.000379	0.559071
Circadian_rhythm	0.000187	0.558208
Natural_killer_cell_mediated_cytotoxicity	0.000156	0.558188
Aminoacyl-tRNA_biosynthesis	4.06E-10	0.557729
Pyrimidine_metabolism	2.26E-06	0.55684
Butanoate_metabolism	2.89E-06	0.556683
Allograft_rejection	0.000367	0.556627
Glucagon_signaling_pathway	0.000573	0.556306
2-Oxocarboxylic_acid_metabolism	6.02E-09	0.556272
Fatty_acid_metabolism	0.000118	0.556181
Transcriptional_misregulation_in_cancer	1.85E-05	0.556067
Melanogenesis	0.000233	0.555755
Glycolysis_Gluconeogenesis	4.10E-05	0.555659
Maturity_onset_diabetes_of_the_young	6.97E-07	0.555127
Notch_signaling_pathway	9.50E-06	0.554771
RNA_degradation	5.21E-07	0.553646
Nicotine_addiction	0.00065	0.553149
ECM-receptor_interaction	0.000513	0.552858

Malaria	1.26E-05	0.552716
Galactose_metabolism	4.17E-08	0.552271
Melanoma	0.000679	0.552252
Lysosome	0.000781	0.550892
alpha-Linolenic_acid_metabolism	3.18E-09	0.549792
Starch_and_sucrose_metabolism	1.89E-05	0.549016
Base_excision_repair	0.00023	0.547704
Fructose_and_mannose_metabolism	0.000327	0.54687
Amino_sugar_and_nucleotide_sugar_metabolism	5.45E-09	0.545852
Histidine_metabolism	0.000392	0.544255
Butirosin_and_neomycin_biosynthesis	0.000508	0.542508
Protein_export	8.51E-06	0.54221
Cardiac_muscle_contraction	2.80E-05	0.536448
Ether_lipid_metabolism	1.86E-05	0.530867
Dilated_cardiomyopathy	0.000549	0.524913
Hypertrophic_cardiomyopathy_(HCM)	0.000608	0.524869
PPAR_signaling_pathway	0.000568	0.519411

Supplementary Table3

Measuring intratumor heterogeneity by network entropy using RNA-seq data

Youngjune Park, Sangsoo Lim, Jin-Wu Nam and Sun Kim

Xenograft data correlation between tITH and number of major clone

KEGG Pathway	Correlation
Glycosphingolipid_biosynthesis	0.940755
Other_types_of_O-glycan_biosynthesis	0.898637
Thyroid_cancer	0.8954
Citrate_cycle_(TCA_cycle)	0.893555
Ribosome	0.889963
Parkinson's_disease	0.886861
Glycosaminoglycan_biosynthesis	0.886838
Butirosin_and_neomycin_biosynthesis	0.874196
Terpenoid_backbone_biosynthesis	0.870596
Protein_export	0.861782
Protein_processing_in_endoplasmic_reticulum	0.850309
mRNA_surveillance_pathway	0.838567
Cell_cycle	0.837966
Oxidative_phosphorylation	0.837536
DNA_replication	0.837356
N-Glycan_biosynthesis	0.825608
ABC_transporters	0.824433
Non-alcoholic_fatty_liver_disease_(NAFLD)	0.822856
Spliceosome	0.819715
Huntington's_disease	0.819138
Insulin_signaling_pathway	0.813044
Carbon_metabolism	0.804002
Alzheimer's_disease	0.80034
Ubiquinone_and_other_terpenoid-quinone_biosynthesis	0.797543
Nucleotide_excision_repair	0.796506
Riboflavin_metabolism	0.795827

Mucin_type_O-Glycan_biosynthesis	0.793335
Other_glycan_degradation	0.789931
Primary_immunodeficiency	0.789037
FoxO_signaling_pathway	0.788088
Propanoate_metabolism	0.785753
T_cell_receptor_signaling_pathway	0.7857
Ovarian_steroidogenesis	0.779908
Maturity_onset_diabetes_of_the_young	0.77945
Proteasome	0.76476
Protein_digestion_and_absorption	0.76322
Acute_myeloid_leukemia	0.761954
D-Glutamine_and_D-glutamate_metabolism	0.761763
Epstein-Barr_virus_infection	0.760006
RNA_transport	0.757161
Metabolic_pathways	0.744838
NOD-like_receptor_signaling_pathway	0.734884
Fatty_acid_metabolism	0.729824
Thyroid_hormone_signaling_pathway	0.72822
Prolactin_signaling_pathway	0.727217
Circadian_rhythm	0.722114
RNA_degradation	0.722048
Proximal_tubule_bicarbonate_reclamation	0.72067
Insulin_resistance	0.714533
Pentose_phosphate_pathway	0.701829
2-Oxocarboxylic_acid_metabolism	0.697318
Cardiac_muscle_contraction	0.692155
Adipocytokine_signaling_pathway	0.689444
Non-small_cell_lung_cancer	0.680639
Basal_transcription_factors	0.674808
Ubiquitin_mediated_proteolysis	0.672935
p53_signaling_pathway	0.671621
ECM-receptor_interaction	0.6706
RNA_polymerase	0.669386
PPAR_signaling_pathway	0.662706
Glycolysis_Gluconeogenesis	0.662485
Butanoate_metabolism	0.661868
Amino_sugar_and_nucleotide_sugar_metabolism	0.660142
Ribosome_biogenesis_in_eukaryotes	0.653694
Natural_killer_cell_mediated_cytotoxicity	0.649047
Central_carbon_metabolism_in_cancer	0.643802
Colorectal_cancer	0.636461
Bile_secretion	0.634361
Pyrimidine_metabolism	0.633991
Sphingolipid_metabolism	0.625771

Phenylalanine_metabolism	0.622046
Renal_cell_carcinoma	0.611568
Folate_biosynthesis	0.604201
B_cell_receptor_signaling_pathway	0.600563
Taurine_and_hypotaurine_metabolism	0.59082
mTOR_signaling_pathway	0.586535
Thiamine_metabolism	0.584694
Salivary_secretion	0.584237
Non-homologous_end-joining	0.574616
Endometrial_cancer	0.568403
Tight_junction	0.568281
Hippo_signaling_pathway	0.563708
Prostate_cancer	0.560112
Starch_and_sucrose_metabolism	0.556205
Oocyte_meiosis	0.550821
Synthesis_and_degradation_of_ketone_bodies	0.549364
Chronic_myeloid_leukemia	0.548331
Endocytosis	0.541776
Notch_signaling_pathway	0.53886
Inflammatory_bowel_disease_(IBD)	0.536678
Phagosome	0.530973
Focal_adhesion	0.527931
Glyoxylate_and_dicarboxylate_metabolism	0.525568
Glycine,_serine_and_threonine_metabolism	0.522503
Primary_bile_acid_biosynthesis	0.521134
Dopaminergic_synapse	0.519699
Mineral_absorption	0.519321
PI3K-Akt_signaling_pathway	0.518572
Caffeine_metabolism	0.516292
Malaria	0.513138
Neurotrophin_signaling_pathway	0.510463
Aldosterone-regulated_sodium_reabsorption	0.487196
Aminoacyl-tRNA_biosynthesis	0.482688
Glioma	0.480063
Dorso-ventral_axis_formation	0.477935
Renin-angiotensin_system	0.476722
HIF-1_signaling_pathway	0.476562
beta-Alanine_metabolism	0.465173
Calcium_signaling_pathway	0.46267
Leukocyte_transendothelial_migration	0.450913
Viral_carcinogenesis	0.447315
Fc_gamma_R-mediated_phagocytosis	0.447035
Nitrogen_metabolism	0.440737
Olfactory_transduction	0.436127

Peroxisome	0.43134
AGE-RAGE_signaling_pathway_in_diabetic_complications	0.428546
Porphyrin_and_chlorophyll_metabolism	0.412105
Pancreatic_secretion	0.402525
Carbohydrate_digestion_and_absorption	0.399834
Fc_epsilon_RI_signaling_pathway	0.397774
Lysine_degradation	0.391736
Glucagon_signaling_pathway	0.387638
VEGF_signaling_pathway	0.383853
AMPK_signaling_pathway	0.381226
Neuroactive_ligand-receptor_interaction	0.380504
Alcoholism	0.378294
Phospholipase_D_signaling_pathway	0.344609
Phenylalanine,_tyrosine_and_tryptophan_biosynthesis	0.344582
Signaling_pathways_regulating_pluripotency_of_stem_cells	0.340166
Ascorbate_and_aldarate_metabolism	0.330573
Choline_metabolism_in_cancer	0.317522
Regulation_of_autophagy	0.29974
cGMP-PKG_signaling_pathway	0.29863
Small_cell_lung_cancer	0.298496
Fatty_acid_elongation	0.296931
Steroid_hormone_biosynthesis	0.296295
Purine_metabolism	0.291484
Transcriptional_misregulation_in_cancer	0.288571
Long-term_depression	0.2833
Cysteine_and_methionine_metabolism	0.282482
Proteoglycans_in_cancer	0.281433
African_trypanosomiasis	0.274089
Staphylococcus_aureus_infection	0.273642
Asthma	0.271417
One_carbon_pool_by_folate	0.259222
Cholinergic_synapse	0.25676
Renin_secretion	0.245688
Cocaine_addiction	0.242389
Melanogenesis	0.238737
Osteoclast_differentiation	0.23843
Pyruvate_metabolism	0.229871
Pancreatic_cancer	0.228873
Adrenergic_signaling_in_cardiomyocytes	0.226157
Insulin_secretion	0.221674
Fatty_acid_degradation	0.218632
Hypertrophic_cardiomyopathy_(HCM)	0.215331
Pathways_in_cancer	0.214923
SNARE_interactions_in_vesicular_transport	0.213672

Hematopoietic_cell_lineage	0.213649
TGF-beta_signaling_pathway	0.212077
Regulation_of_lipolysis_in_adipocytes	0.211786
Biosynthesis_of_unsaturated_fatty_acids	0.203137
Hepatitis_B	0.194702
MicroRNAs_in_cancer	0.18327
ErbB_signaling_pathway	0.17664
Biotin_metabolism	0.17258
Vasopressin-regulated_water_reabsorption	0.16846
MAPK_signaling_pathway	0.152152
Shigellosis	0.151748
Amphetamine_addiction	0.147335
Tuberculosis	0.132774
Vitamin_digestion_and_absorption	0.130224
Chagas_disease_(American_trypanosomiasis)	0.126222
Type_II_diabetes_mellitus	0.119724
Bacterial_invasion_of_epithelial_cells	0.11818
Estrogen_signaling_pathway	0.113375
Complement_and_coagulation_cascades	0.111294
Sphingolipid_signaling_pathway	0.098962
Melanoma	0.097176
Platelet_activation	0.091386
Pertussis	0.080096
Bladder_cancer	0.078838
Hepatitis_C	0.07054
Aldosterone_synthesis_and_secretion	0.069054
Circadian_entrainment	0.068873
Toxoplasmosis	0.068092
Axon_guidance	0.067047
Wnt_signaling_pathway	0.063613
Thyroid_hormone_synthesis	0.055527
Regulation_of_actin_cytoskeleton	0.042105
Mismatch_repair	0.042044
GnRH_signaling_pathway	0.037739
Taste_transduction	0.027163
Histidine_metabolism	0.02695
TNF_signaling_pathway	0.024764
Metabolism_of_xenobiotics_by_cytochrome_P450	0.021511
Steroid_biosynthesis	0.020126
Cell_adhesion_molecules_(CAMs)	0.014778
Progesterone-mediated_oocyte_maturation	0.009273
Alanine,_aspartate_and_glutamate_metabolism	0.008506
Chemical_carcinogenesis	0.005602
Dilated_cardiomyopathy	0.004517

cAMP_signaling_pathway	-0.00222
Cytokine-cytokine_receptor_interaction	-0.00239
Chemokine_signaling_pathway	-0.00668
Tyrosine_metabolism	-0.01549
Rap1_signaling_pathway	-0.0204
Base_excision_repair	-0.0235
Glutamatergic_synapse	-0.02655
Measles	-0.04763
Amyotrophic_lateral_sclerosis_(ALS)	-0.07743
Antigen_processing_and_presentation	-0.07876
Morphine_addiction	-0.08066
Legionellosis	-0.08328
Endocrine_and_other_factor-regulated_calcium_reabsorption	-0.09334
Arginine_and_proline_metabolism	-0.09965
Basal_cell_carcinoma	-0.10148
Degradation_of_aromatic_compounds	-0.10201
GABAergic_synapse	-0.10246
Oxytocin_signaling_pathway	-0.10327
Lysosome	-0.14276
Vibrio_cholerae_infection	-0.17978
Retinol_metabolism	-0.18189
Epithelial_cell_signaling_in_Helicobacter_pylori_infection	-0.18316
Retrograde_endocannabinoid_signaling	-0.18904
HTLV-I_infection	-0.24934
Arrhythmogenic_right_ventricular_cardiomyopathy_(ARVC)	-0.2509
Phototransduction	-0.26034
Ras_signaling_pathway	-0.26837
Influenza_A	-0.2712
Fat_digestion_and_absorption	-0.29984
Gastric_acid_secretion	-0.30423
Pathogenic_Escherichia_coli_infection	-0.30976
Amoebiasis	-0.31746
Nicotine_addiction	-0.31937
Fatty_acid_biosynthesis	-0.32793
Sulfur_metabolism	-0.34093
Collecting_duct_acid_secretion	-0.35524
Serotonergic_synapse	-0.35664
Jak-STAT_signaling_pathway	-0.35717
Systemic_lupus_erythematosus	-0.3698
Leishmaniasis	-0.38091
Glycerophospholipid_metabolism	-0.38445
Vascular_smooth_muscle_contraction	-0.39226
Biosynthesis_of_amino_acids	-0.39459
alpha-Linolenic_acid_metabolism	-0.39635

Arginine_biosynthesis	-0.3966
Autoimmune_thyroid_disease	-0.40767
Ether_lipid_metabolism	-0.40927
Synaptic_vesicle_cycle	-0.4169
Pentose_and_glucuronate_interconversions	-0.42335
Selenocompound_metabolism	-0.42636
Salmonella_infection	-0.43017
RIG-I-like_receptor_signaling_pathway	-0.43259
Cyanoamino_acid_metabolism	-0.43405
Tryptophan_metabolism	-0.43441
Pantothenate_and_CoA_biosynthesis	-0.43764
Gap_junction	-0.4454
Valine,_leucine_and_isoleucine_degradation	-0.45785
Prion_diseases	-0.46297
Inflammatory_mediator_regulation_of_TRP_channels	-0.46697
Inositol_phosphate_metabolism	-0.51161
Long-term_potentiation	-0.52951
Intestinal_immune_network_for_IgA_production	-0.54055
Apoptosis	-0.543
Cytosolic_DNA-sensing_pathway	-0.55091
Viral_myocarditis	-0.57345
Phosphatidylinositol_signaling_system	-0.57793
Herpes_simplex_infection	-0.5894
Drug_metabolism	-0.59088
Toll-like_receptor_signaling_pathway	-0.59272
Homologous_recombination	-0.62655
Linoleic_acid_metabolism	-0.63239
NF-kappa_B_signaling_pathway	-0.63378
Glutathione_metabolism	-0.64568
Allograft_rejection	-0.67149
Glycerolipid_metabolism	-0.6887
Glycosaminoglycan_degradation	-0.69246
Galactose_metabolism	-0.69836
Sulfur_relay_system	-0.72322
Graft-versus-host_disease	-0.72397
Hedgehog_signaling_pathway	-0.72967
Fructose_and_mannose_metabolism	-0.73444
Rheumatoid_arthritis	-0.76823
Adherens_junction	-0.77763
Vitamin_B6_metabolism	-0.78391
Arachidonic_acid_metabolism	-0.84811
Nicotinate_and_nicotinamide_metabolism	-0.85898
Glycosylphosphatidylinositol(GPI)-anchor_biosynthesis	-0.87017
Type_I_diabetes_mellitus	-0.87078

Fanconi_anemia_pathway	-0.94469
